# Contribution of Ambient Ozone to Changes in Scots Pine Defoliation. Step II of Lithuanian Studies

**DOI:** 10.1100/tsw.2007.54

**Published:** 2007-03-21

**Authors:** Algirdas Augustaitis, Ingrida Augustaitiene, Almantas Kliucius, Rasele Girgzdiene, Dalia Sopauskiene

**Affiliations:** ^1^ Lithuanian University of Agriculture, LT-53362 Kaunas dstr., Lithuania; ^2^ Institute of Physics, Savanoriu 231, LT-02300, Vilnius, Lithuania

**Keywords:** ozone, acidifying compounds, acid deposition, mean temperature, amount of precipitation

## Abstract

This study aimed to explore if changes in peak ozone (O_3_) concentrations may reinforce the phytotoxic effects of air concentration of acidifying compounds and their deposition, as well as unfavorable climatic factors on pine crown defoliation. Forty-eight pine stands with more than 8000 sample pine trees have been monitored annually. The impact of sulfur dioxide (SO_2_) on pine defoliation was found to be the most significant. The impacts of peak O_3_ concentrations, acid deposition, and amount of precipitation were considerably lower, whereas the impact of air temperature, the least. Contribution of peak O_3_ concentrations to the integrated impact of acid deposition and amount of precipitation on pine defoliation was most significant, whereas the contribution to the impact of acidifying air compounds, mainly SO_2_, was the least. No synergetic effect between peak O_3_ concentrations and high temperature during vegetation period was detected.

### INTRODUCTION

Ambient ozone ($O_3$) is considered to be one of the most important and pervasive phytotoxic agents whose effects are likely to increase in the future[1,2,3]. Its effects on plants are the result of a three-step chain of events: exposure, uptake, and biological effect[4]. The most important is stomatal $O_3$ uptake, which provides a more reliable indicator for the assessment of risk to plants[5,6] because only the amount of $O_3$ arriving at the cell membrane or, perhaps, at the cell wall (apoplast) can have a biological effect on the tissue[4,7]. In this way, $O_3$ can cause a wide range of symptoms, the most important of which are as follows: decrease in foliar chlorophyll content and necrotic spots[8,9,12], acceleration of leaf senescence and reduction in leaf life span[7,10,11], photosynthesis[12,13,14], growth and productivity[15,16,17,18]. Leaf and needle drop, or reduction in foliage biomass (defoliation), was chosen as the main object of the presented studies in order to explore the possible effect of $O_3$ on pine trees in natural forest ecosystems.

In this context, many scientists are realizing that acid rain and ambient $O_3$ are the key factors resulting in spatial and temporal changes of tree crown defoliation[5,19,20,21]. Their combined effects differ significantly from a sum of separate effects due to their synergistic or antagonistic interactions[23]. Most likely, therefore, it is stated that the process base of $O_3$ damage to plants is still not fully clarified[6,24], and the relationship between forest tree crown condition and $O_3$ is not well established[18,22,25,26] and often fails[26,27]. Therefore, most of the authors investigating $O_3$ effect on forests suggested studying $O_3$ impact in the context of other environmental factors[28]

In our earlier studies[29,30], we stated that air concentration of the acidifying compounds, their concentration in precipitation, and wet deposition were key factors resulting in approximately 60% of pine defoliation variability in the areas under regional pollution in Lithuania. Meteorological factors increased the degree of explanation by 7%[31]. If this is true, the effect of $O_3$ on pine defoliation should be significant as well because its concentration over the considered period was closest to critical if compared with air concentration of sulfur and nitrogen compounds and their deposition.

In our first step of the presented studies, peak $O_3$ concentration was chosen as the most acceptable index to detect possible effect of $O_3$ on biota. In this study, instead of the possible effect of $O_3$ on mean station-wise values of defoliation (degree of freedom 28), we studied the possible effect on stand-wise mean values of Scots pine (*Pinus sylvestris* L.) crown defoliation from additional dataset with degree of freedom more than 400. This remarkable increase in degree of freedom enabled us to investigate the possible effect of all considered natural and anthropogenic environmental parameters on pine defoliation and quantify contribution of $O_3$ to the integrated impact of other environmental factors on pine defoliation. A working hypothesis was that in natural forest ecosystems, changes in pine defoliation are related to changes in peak $O_3$ concentrations that reinforce the effects of other natural and anthropogenic stresses.

### MATERIALS AND METHODS

#### Response Variables

To get more insight into the relationship between different $O_3$ indices and pine defoliation, annual defoliation data of 48 pine stands located close to the Integrated Monitoring Stations (IMS) available for a 12-year period in Aukstaitija and Zemaitija National Parks (NP) and 6-year period (1994–1999) in Dzukija NP were used as a response variable. These stands were selected according to stand maturity: 8 sapling stands (45–50 years), 10 middle-aged stands (61–80 years), 11 premature stands (81–100 years), 11 mature stands (101–120 years), and 8 over-mature stands (>121 years).

Data on tree and stand parameters (mean diameter and height of trees, tree density, basal area, and tree volume per hectare), as well as crown defoliation, were available from the permanent observation stands. A three-stage sampling pattern was used for the collection of the field material: (1) sampling of the research stands, (2) sampling of circular plots within each research stand, (3) sampling of trees for more detailed measurements of the tree crown parameters and tree ring analysis (these data are included in step III of our study)[32]. Each pine stand included 12 circular sample plots with an average of 15–20 trees in each plot. The sample plots were distributed systematically in a grid system. Crown defoliation of more than 8000 pine trees was assessed annually at the end of August through the beginning of September. The ICP Forest Monitoring Methodology was employed to assess tree defoliation[33].

#### Predictor Variables and Methods of Their Estimation

Thirty considered predictor variables were classified into three groups. The first group included three site-specific variables, i.e., forest type, soil typological groups with respect to fertility and moisture conditions, and five tree and stand variables: mean tree age, height, diameter, basal area, and stand volume. The second group consisted of 10 meteorological variables (air temperature and amount of precipitation of eight seasons of 2 years and two year-long periods from September to August). The third group of 13 variables included data on air concentrations of sulfur dioxide ($SO_2$), sulfate ($SO_4^{2-}$), the sum of nitrate compounds ($\Sigma NO_3^- = NO_3^- + HNO_3$), and the sum of ammonium compounds ($\Sigma NH_4^+ = NH_4^+ + NH_3$); concentration of $SO_4^{2-}$, $NO_3^-$, $NH_4^+$, and $H^+$ in precipitation as well as their wet deposition. Continuous monitoring of gaseous pollutants with active samplers was conducted based on EMEP methodology[34].

### Method of the Study

The integrated impact of natural and anthropogenic factors on mean defoliation of pine trees was analyzed by a multiple stress approach, using the linear multiple regression technique of "Statistica 6.0" software. The quality of the created models was assessed by determining the coefficient of determination ($r^2$) and the level of statistical significance ($p$). Stress factors were excluded from the regression model by a stepwise procedure based on the level of significance of each stress factor. Finally, variables with a high level of significance compiled the models.

The impact of ambient $O_3$ on pine crown defoliation was examined in a two-step multiregression procedure[35]. In a first step, the annual mean defoliation was regressed on site and stand predictor variables using a stepwise regression with a forward selection procedure. Then, the residual defoliation was regressed on air concentrations of acidifying compounds, their concentration in precipitation and deposition, and meteorology using the same stepwise regression procedure. Finally, predictor variable "$O_3$" was included in a created model and its contribution to the integrated impact of natural and anthropogenic factors on pine crown defoliation was quantified.

## RESULTS

### Changes in Ambient $O_3$ and Acidifying Air Compounds and Their Deposition

Data available from IMS indicated a significant decrease in air concentrations of sulfur compounds and ammonia, and their deposition until 2000 (Fig. 1). Afterwards, stabilization or some increase was observed. Changes in air concentration of $NO_3^-$ and its deposition had no significant trend. However, since 2001, a tendency towards an increase in air concentration of $NO_3^-$ was detected. The detected changes in peak $O_3$ concentration and AOT40 index for forests were similar to changes in air concentration of $SO_2$, $\Sigma NH_4^+$ and $NO_3^-$ deposition (Step I of Lithuanian studies).

### Temporal and Spatial Changes in Meteorology

The annual amount of precipitation differed significantly ($p < 0.05$) among national parks. The mean annual precipitation was 930 mm at Zemaitija NP, 710 mm at Aukstaitija NP, and 650 mm at Dzukija NP. The precipitation amount from October through February accounted for these differences. However, there were no significant temporal differences in annual precipitation during the 1994–2005 period. The average amount of precipitation during the autumn and winter seasons was stable, while in spring it decreased by 10.4 mm per season ($p < 0.037$) and in summer increased by 13.3 mm per season ($p < 0.010$).

The mean annual temperature tended to increase between 1994 and 2005 ($p > 0.05$). At Aukstaitija, an average increase made 0.013ºC/year, at Dzukija 0.061ºC, and at Zemaitija 0.069ºC. The increase was most pronounced in autumn (September–November). The mean annual temperature in Zemaitija (+6.9ºC) was significantly higher than in Aukstaitija (+6.4ºC) and Dzukija (+6.5ºC) ($p < 0.05$).

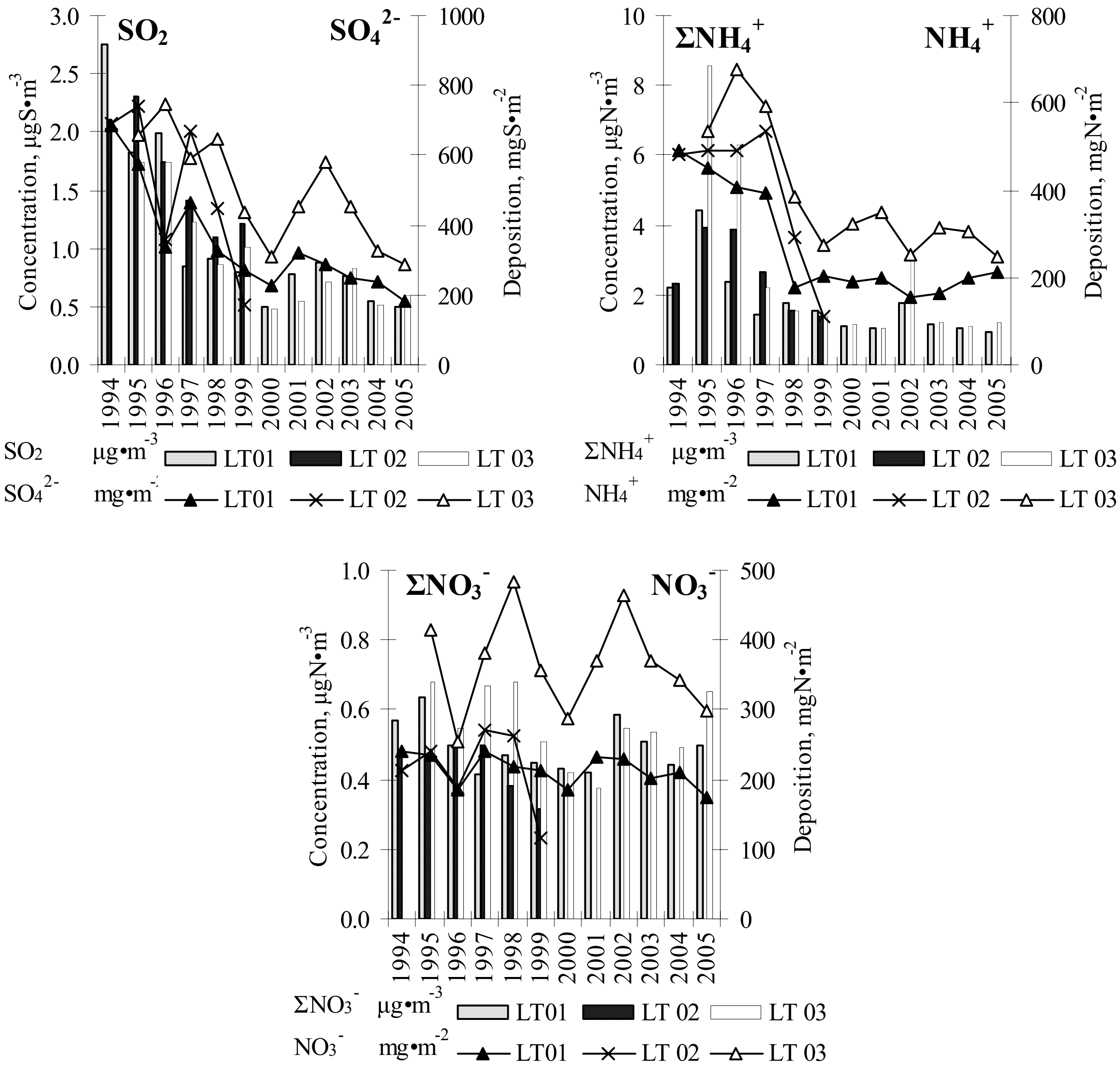

**FIGURE 1.** Air concentrations of the considered pollutants (▒) and their deposition (^).

### Temporal and Spatial Changes in Mean Defoliation of Scots Pine Trees

Scots pine trees in Aukstaitija NP showed the best condition as illustrated in Fig. 2. On the poorest sites (*Pinetum cladoniosum* forest type) in Dzukija NP, outbreaks of forest pests (*Diprion pini L.* and *Ocneria monacha L.)* started in 1992 after a hot and dry vegetation period in 1991 and caused very serious crown damage. Long-term elevated levels of the atmospheric N and S depositions and $O_3$ concentrations could have had additional effect on tree defoliation in this park. In 1996, biological insecticide Foray-48B was applied to suppress the outbreak and recovery of the damaged Scots pine trees started.

The highest level of mean defoliation of pine trees in Aukstaitija and Dzukija NPs was observed in 1995, whereas in Zemaitija NP it was seen in 1997 (Fig. 2). Afterwards, crown condition showed obvious improvement that lasted until 2000–2001. Between 2002–2005, tree condition deteriorated again. These detected temporal changes in mean defoliation of pine trees were common for most of Europe[36].

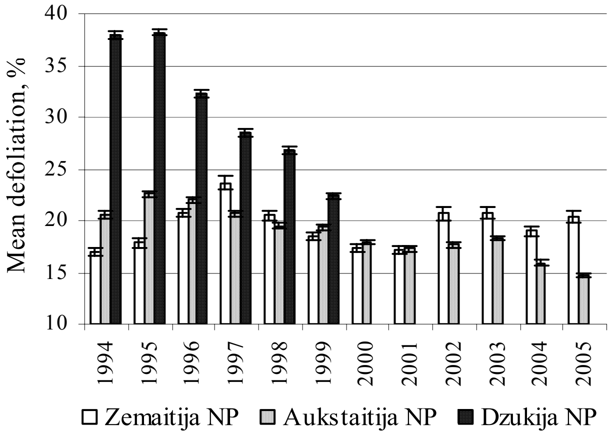

**FIGURE 2.** Mean value and standard errors of pine defoliation in Lithuanian national parks.

### Changes in Pine Defoliation in Relation to Concentrations of the Considered Pollutants

In Aukstaitija NP, correlation coefficient between mean stand-wise defoliation and peak $O_3$ concentration in 13 of 22 stands was statistically significant ($p < 0.05$, $r > 0.56$); in Dzukija NP in 5 of 16 stands ($r > 0.80$) and in Zemaitija NP in 3 of 9 stands (Table 1). Main statistics of stand-wise correlative coefficients are compiled in Table 1. The changes in mean stand-wise defoliation of pines are most likely related to changes in acidifying compounds and peak concentration of ambient ozone.

**TABLE 1**
**The Main Statistics of Correlation Coefficients between Stand-wise Defoliation and Considered Pollution Variables**

| Parameter | Ozone | | | | Air Concentration | | | Deposition | | |
|---|---|---|---|---|---|---|---|---|---|---|
| | Mean | IV–VIII | Peak | AOT40 | $SO_2$ | $\sum NH_4^+$ | $\sum NO_3^-$ | $SO_4^{2-}$ | $NH_4^+$ | $NO_3^-$ |
| Aukstaitija NP | | | | | | | | | | |
| Mean | 0.001 | 0.165 | 0.552 | 0.384 | 0.588 | 0.626 | 0.306 | 0.583 | 0.598 | 0.411 |
| Min | –0.325 | –0.170 | 0.211 | 0.091 | –0.026 | 0.290 | –0.237 | 0.143 | 0.213 | 0.102 |
| Max | 0.419 | 0.412 | 0.782 | 0.768 | 0.924 | 0.867 | 0.629 | 0.916 | 0.921 | 0.685 |
| Std. dev. | 0.214 | 0.180 | 0.188 | 0.143 | 0.261 | 0.145 | 0.213 | 0.231 | 0.232 | 0.150 |
| Dzukija NP | | | | | | | | | | |
| Mean | –0.205 | –0.185 | 0.707 | 0.310 | 0.831 | 0.688 | –0.165 | 0.437 | 0.712 | –0.580 |
| Min | –0.626 | –0.637 | 0.463 | –0.068 | 0.574 | 0.316 | –0.452 | 0.276 | 0.239 | –0.705 |
| Max | 0.261 | 0.348 | 0.837 | 0.532 | 0.965 | 0.912 | 0.340 | 0.617 | 0.858 | –0.276 |
| Std. dev. | 0.271 | 0.304 | 0.106 | 0.199 | 0.130 | 0.223 | 0.204 | 0.087 | 0.143 | 0.119 |
| Zemaitija NP | | | | | | | | | | |
| Mean | 0.210 | 0.150 | 0.275 | –0.147 | 0.123 | –0.031 | 0.342 | 0.250 | 0.235 | 0.080 |
| Min | –0.244 | –0.315 | –0.422 | –0.582 | –0.391 | –0.318 | –0.370 | –0.268 | –0.581 | –0.329 |
| Max | 0.635 | 0.564 | 0.757 | 0.403 | 0.846 | 0.676 | 0.749 | 0.822 | 0.885 | 0.347 |
| Std. dev. | 0.243 | 0.284 | 0.376 | 0.264 | 0.388 | 0.316 | 0.386 | 0.297 | 0.429 | 0.227 |

*Note:* Std.dev. – Standard deviation.

The air concentrations of $SO_2$ and $NH_4^+$, as well as their wet deposition, showed the strongest statistically significant relationships with temporal and spatial changes in mean defoliation of Scots pines ($p < 0.05$; n = 421) (Fig. 3). The obtained results are in agreement with the ICP Forests Monitoring data[37,38,39]. Correlation coefficient between peak $O_3$ concentration and temporal and spatial changes in mean pine defoliation was weaker. Significance of AOT40-2 for forest was twofold lower; meanwhile AOT40-1 for vegetation as well as other considered $O_3$ indices were not significant ($p > 0.05$).

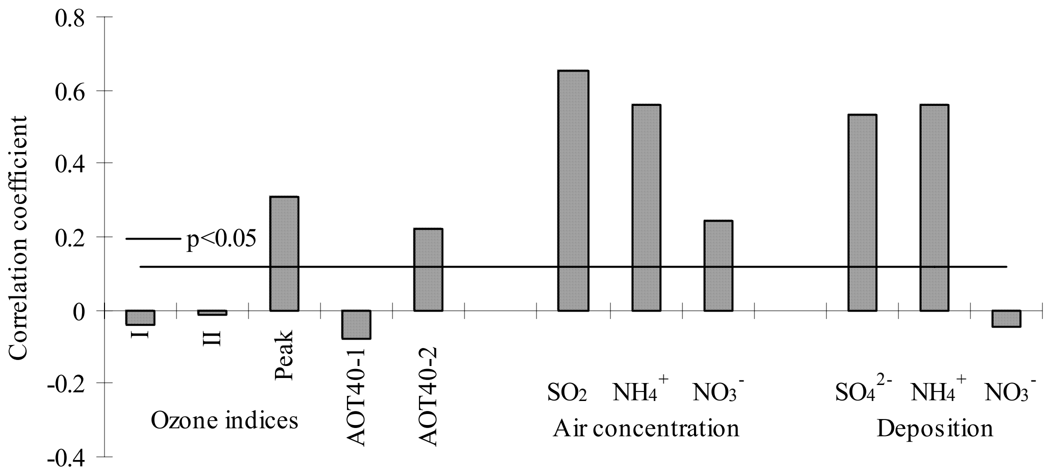

**FIGURE 3.** Correlation coefficients of mean defoliation of pine stands and considered pollution variables in Lithuanian national parks (I – mean annual value; II – mean value for vegetative period; AOT40-1 for vegetation; AOT40-2 for forest)

### Integrated Impact of the Natural and Anthropogenic Environmental Factors on Defoliation

Correlation between pine defoliation and stand density was found to be strongest (r = 0.38), followed by a weaker negative correlation with stand volume (r = –0.23) and age (r = –0.19, $p < 0.05$). Significant differences in defoliation were established among stands from different sites. The highest mean defoliation (36.4% ± 1.0) was recorded on *Pinetum cladoniosum* forest type (FT), the lowest on *P. vacciniosum* FT (19.2% ± 0.2) and *P. oxalidosum* FT (19.6% ± 1.4). These data revealed that stand and site parameters had significant effect on spatial distribution of pine defoliation. Integrated impact of these parameters accounted for more than 60% variation in defoliation:

$$F = 11.61 + 1.378 \times A - 0.169 \times \sum G + 0.012 \times N + 1.884 \times FType; \; R^2 = 0.610, \; p < 0.05 \quad (1)$$

F = crown defoliation, %; A = stand age, years; $\sum G$ = sum of tree basal area, $m^2$ per ha; N = tree number, units/ha; FType = forest type (categorical value).

Residual part of the unexplained defoliation was regressed with air pollutants, acid deposition, and meteorological parameters. The strongest relationship was detected between air concentration of $SO_2$ and pine defoliation residual (r = 0.50), followed closely by the relationship between peak $O_3$ concentration and defoliation residual (r = 0.44). There was no synergetic effect of $O_3$ and $SO_2$ on pine defoliation; peak $O_3$ values reinforce the effect of $SO_2$ on residuals by only 2.4% (Table 2). However, $O_3$ reinforced the impact of acid deposition and drought on pine defoliation more significantly, by approximately 6%, and mean temperature of dormant period by 11%. In our case, when impacts of all considered predictor variables were accounted, the effect of peak $O_3$ values remained significant ($p < 0.05$), increasing the degree of explanation of defoliation residuals variability up to 30%. These results verify the hypothesis

**TABLE 2**
**Contribution of the $O_3$ to Integrated Impact of Different Environmental Factors on Residuals of Pine Crown Defoliation**

| Variables | Models, F(a.b) | | | | | | | | |
|---|---|---|---|---|---|---|---|---|---|
| | **(1.419)** | **(2.418)** | **(2.418)** | **(3.417)** | **(4.416)** | **(2.418)** | **(2.418)** | **(5.415)** | **(6.414)** |
| In the air: | | | | | | | | | |
| $SO_2$ | + | | | | | | | | + |
| $\sum NH_4^+$ | | | | | | | + | | |
| $\sum NO_3^-$ | | | | | | + | | | |
| In precipitation: | | | | | | | | | |
| $SO_4^{2-}$ | | + | | | | | | | + |
| $NH_4^+$ | | + | | | | | | | |
| $NO_3^-$ | | | | | | | | | |
| Deposition: | | | | | | | | | |
| $SO_4^{2-}$ | | | + | | | | | | |
| $NH_4^+$ | | | | | | | + | | |
| $NO_3^-$ | | | + | | | + | | | |
| Precipitation: | | | | | | | | | |
| Last season: | | | | | | | | | |
| IX–XI | | | | | | | | | |
| XII–II | | | | | | | | + | + |
| III–V | | | | | | | | | |
| VI–VIII | | | | + | | | | + | + |
| Current season: | | | | | | | | | |
| IX–XI | | | | + | | | | | |
| XII–II | | | | | | | | | |
| III–V | | | | | | | | | |
| VI–VIII | | | | + | | | | + | |
| Temperature: | | | | | | | | | |
| Last season: | | | | | | | | | |
| IX–XI | | | | | + | | | | |
| XII–II | | | | | + | | | + | + |
| III–V | | | | | | | | | |
| VI–VIII | | | | | | | | | |
| Current season: | | | | | | | | | |
| IX–XI | | | | | + | | | + | |
| XII–II | | | | | + | | | | |
| III–V | | | | | | | | | |
| VI–VIII | | | | | | | | | |
| $r^2$ , % | 25.4 | 18.1 | 17.3 | 20.0 | 11.5 | 7.1 | 18.0 | 24.0 | 29.0 |
| $r^{2*}$ with $O_3$ effect, % | 27.7 | 26.1 | 23.2 | 25.7 | 22.9 | 19.5 | 25.8 | 26.7 | 29.9 |
| $O_3$ effect ($r^{2*} - r^2$), % | 2.4 | 8.0 | 5.9 | 5.7 | 11.4 | 12.4 | 7.8 | 2.7 | 1.0 |
| $O_3$ significance: $p <$ | 0.000 | 0.000 | 0.000 | 0.000 | 0.000 | 0.010 | 0.010 | 0.000 | 0.034 |

*Note:* Individual impact of peak $O_3$ concentration on defoliation residual: $r^2 = 19.3\%$ and $p < 0.0001$; F(a.b), models identified by F - test symbol with numbers of degrees of freedom: a, of the predictor variables; b, of the observations (STATISTICA software).

that in regions affected by air pollution, peak $O_3$ values are most likely among the key factors affecting spatial and temporal variability of Scots pine defoliation.

### DISCUSSION

To date, most scientists agree that forest condition is influenced by a multitude of different stress factors[37,38,39]. Therefore, the effect of air pollution, including $O_3$, could in principle only be estimated properly if all other relevant effects are taken into account, simultaneously including them into a multivariate statistical analysis[6]; what often helps in decoding the role of different predictors[40]. However, in this way, an attempt to account all possible predictor variables could be impossible due to too high a number of variables. The maximum number of degrees of freedom of the predictor variables should not exceed 25% of the number of observations, and should even be lower when temporal and spatial correlation between them is expected[41]. Therefore, to improve detection of the significance of key factors, the possible impact of predisposing factors was accounted.

Highly significant relations were derived between defoliation and stand age, tree, and site-specific parameters, such as soil moisture and nutrient content[27,38,39,42,43]. These parameters cannot directly affect tree condition, however, their predisposing effects are well known. In case of ignoring their effect, definition of key stress-effect relationship often failed[27]. Therefore, in order to meet the objectives of the present study, relationships between pine defoliation and pollution and meteorology were examined after the influences of site and stand parameters had been accounted. This procedure could be evaluated as successful because significance of the relationships between defoliation residuals, and meteorology, and acidifying compounds remained at the same level, whereas significance of the relationship between defoliation residuals and peak $O_3$ concentrations increased considerably.

Despite the statements that below the phytotoxic level no direct threat to vegetation from $SO_2$[44,45] or synergetic interaction between $SO_2$ and $O_3$ could be expected[46,47], the presented data confirm our earlier findings that acidifying air compounds and their deposition are the key factors resulting in pine defoliation changes[29,30]. They could explain from 23–28% variance of residual defoliation of pine trees. The effect of peak $O_3$ concentrations was less significant (19.3%), however, the presented data verified the statement that $O_3$ could reinforce the effects of other air pollutants such as SOx and NOx[19,20]. Ozone peak concentrations increase the explanation rate of defoliation residual variability by air concentration of acidifying compounds, their concentration in precipitation, and deposition by almost 3–8%.

Drought, especially during the vegetation period, is often mentioned as one of the key factors resulting in defoliation changes. McLaughlin and Downing found that the effects of $O_3$ are synergistic with high temperature and moisture stress[48]. However, there are contrary statements indicating that the effect of $O_3$ and drought might counterbalance each other[6]. Closed stomata protect foliage from the highest concentrations of $O_3$, which are characteristic for this period. This contrary interaction could be explained by the fact that despite increase in $O_3$ concentrations from north towards south[17,49], $O_3$ exposure in northern latitudes often leads to plants becoming more susceptible to injury than in southern areas[50]. Long, bright days and high humidity in air and soil are typical for the situation in large parts of the Nordic countries[49]. The peak concentrations are typical in spring[14]. Not very high air temperature, low vapor pressure deficits, and sufficient soil water supply is characteristic for this period. Therefore, in these areas, $O_3$ flux inside the leaves could be higher if compared with southern areas.

Generalizing the presented results, we could conclude that $O_3$ peak values could be considered as one of the key factors affecting pine crown condition in Lithuania.

### CONCLUSIONS

Correlation coefficient between temporal and spatial changes in the peak ambient $O_3$ concentrations and changes in mean defoliation of Scots pine trees in Lithuania is statistically significant. However, the significance is lower than it is between defoliation and the $SO_2$ air concentration, approximately the same as between defoliation and the acidifying compounds in precipitation, acid deposition, and amount of precipitation, but considerably higher than between defoliation and mean air temperature. When the effect of air acidifying compounds, their deposition, and climatic factors was accounted, the contribution of peak concentrations of ambient $O_3$ to pine defoliation changes remained significant increasing the general rate of explanation of the spatial and temporal defoliation variance.
